# Proteomic and miRNA Profiles of Exosomes Derived from Myometrial Tissue in Laboring Women

**DOI:** 10.3390/ijms232012343

**Published:** 2022-10-15

**Authors:** Wenfeng Deng, Xiaodi Wang, Lina Chen, Bolun Wen, Yunshan Chen, Kaiyuan Ji, Huishu Liu

**Affiliations:** 1 Guangzhou Key Laboratory of Maternal-Fetal Medicine, Department of Obstetrics and Gynecology, Guangzhou Women and Children’s Medical Center, Guangzhou Medical University, Guangzhou 510623, China; 2 School of Medicine, South China University of Technology, Guangzhou 510006, China

**Keywords:** exosomes, myometrium, proteomics, miRNA-seq

## Abstract

Myometrial contraction is essential for successful delivery. Recent studies have highlighted the vital roles of tissue-derived exosomes in disease diagnostic, prognostic, and therapeutic applications; however, the characteristics of uterine myometrium-derived exosomes are unclear. Here, we successfully isolated exosomes from myometrial tissues, human myometrial smooth muscle cells (HMSMCs), and human umbilical vein endothelial cells (HUVECs), then performed quantitative liquid chromatography-tandem mass spectrometry and miRNA sequencing to investigate the cargo of the exosomes. Fifty-two proteins and five miRNAs were differentially expressed (DE) in term non-labor and term labor myometrium-derived exosomes. Among them, seven proteins (SERPINE1, THBS1, MGAT1, VIM, FGB, FGG, and VWF) were differentially expressed both in the myometrial exosomes and tissues, three miRNAs (miR-363-3p, miR-203a-3p, and miR-205-5p) target 13 DE genes. The top three miRNA derived from HMSMCs (miR-125b-1-3p, miR-337-5p, and miR-503-5p) and HUVECs (miR-663a, miR-4463, and miR-3622a-5p) were identified. Two proteins, GJA1 and SLC39A14, exist in female blood exosomes and are highly expressed in HMSMCs exosomes, are also upregulated in the laboring myometrium, which verified increased in laboring blood samples, might be novel potential biomarkers for myometrial activation. The proteomic and miRNA profile of exosomes derived from laboring myometrium revealed some molecules in the exosomes that affect the intercellular communication and the function of the myometrium.

## 1. Introduction

Exosomes are extracellular vesicles with a diameter of 40–150 nm, consisting of a bilayer plasma membrane and containing molecular components of their source cells, including immunosuppressive and immunostimulatory proteins, chemokines, cytokines, cell receptors, lipids, and RNAs [1,2,3,4]. These compounds convey paracrine signals between tissues and are closely associated with signal transduction between cells.

Compared with the exosomes derived from cell supernatants or body fluids, the exosomes isolated directly from the tissues possess reflection of tissue specificity and tissue microenvironment. Recent studies have demonstrated that tissue-exosomes were associated with the biogenesis and evolution of various cancer events, such as angiogenesis, local invasion, and distant metastasis [5]. Exosomes could participate in the development of multiple non-cancer diseases, including cardiac and cerebral ischemia/reperfusion injury [6,7]. Increasing attention has been paid to the clinical application of exosomes, as potential markers for diagnosis, prognosis, and therapeutic applications. A study on tissue-derived exosomes [8] showed that the most abundant protein in bladder tissue-derived exosomes, regardless of the site, was enriched in metabolic carcinogenesis-related pathways and was associated with a poor prognosis, which supports the notion of urinary exosomes as potential biomarkers for urinary bladder cancer. We investigated whether the myometrium-derived exosomes might contain certain markers that could characterize the functional status of the uterine muscles.

During pregnancy, extracellular vesicles are essential for fetal–maternal crosstalk, and exosomes are considered necessary for successful embryonic implantation and normal fetus development [9]. Moreover, exosomes can be potential biomarkers of maternal complications such as preterm birth, fetal growth restrictions, preeclampsia, and gestational diabetes mellitus [10,11,12]. Exosomes participate in contractions of skeletal muscle and vascular smooth muscle cells [13,14,15]. Uterine contraction during labor is a fundamental physiological activity, which is a complex process. Our previous study showed [16] that the transcriptome and proteome of myometrium changed markedly during labor, especially affecting the functions of inflammation and hypoxia. However, the roles of myometrial exosomes remain to be studied.

In this study, we expand the characterization of exosomes isolated from human laboring myometrium, human myometrial smooth muscle cells (HMSMCs), and human umbilical vein endothelial cells (HUVECs), exploring the proteomic and miRNA characteristics of these exosomes to investigate the HMSMCs- and HUVECs-specifically secreted cargoes in the myometrium, and to explore the novel biomarkers for myometrial contraction.

## 2. Results

### 2.1. Characterization of Exosomes Isolated from Human Myometrial Tissue, HMSMCs, and HUVECs

**Figure 1:**
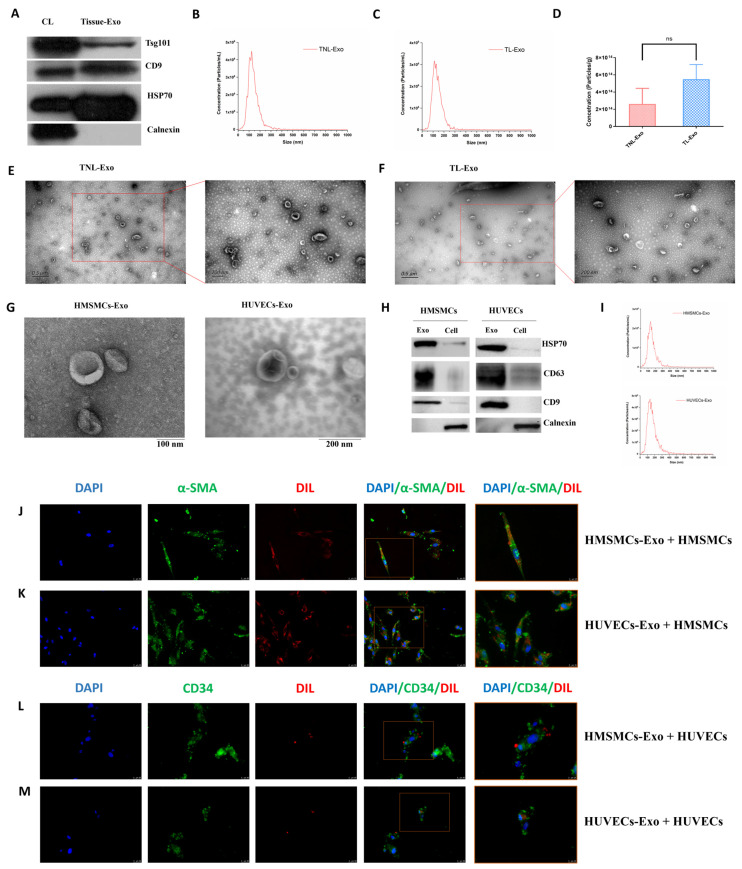
Characterization of exosomes isolated from myometrial tissue, HMSMCs and HUVECs. (**A**) Western blotting for Tsg101, CD9, HSP70, and Calnexin in cell lysis (CL) and myometrial tissue exosomes (Tissue-Exo). (**B**,**C**) Nanoparticle tracking analysis (NTA) measurements showing the size distribution of exosomes in term non-labor (TNL) and term labor (TL) myometrial exosomes. (**D**) Concentrations of exosomes derived from TNL and TL myometrial tissues had no significantly difference (*p* > 0.05). (**E**–**G**) TEM showing round/cup-shaped exosomes in the myometrial, HMSMC, and HUVEC exosomes. (**H**) Western blotting for HSP70, CD63, CD9, and Calnexin in the HMSMC and HUVEC exosomes. (**I**) NTA showing the size distribution of HMSMC and HUVEC exosomes. (**J**–**M**) Absorbing of Dil-labelled HMSMC and HUVEC exosomes inside the cells. DAPI was used to stain nuclear and α-SMA/CD34 were used to stain HMSMCs/HUVECs, respectively. Exo, exosomes. ns, not significant.

Human myometrial tissue exosomes contained exosome markers Tsg101, CD9, and HSP70, without the negative marker calnexin (Figure 1A), indicating that the exosomes were representative. Exosomes showed a typical size distribution and morphology (Figure 1B,C). There were no significant differences in myometrial-derived exosome concentrations between non-labor and labor (Figure 1D). Transmission electronic microscopy (TEM) of exosomes isolated from myometrial tissue samples, HMSMCs, and HUVECs showed round, cup-shaped exosomes (Figure 1E–G). HMSMC and HUVEC exosomes were shown in a previous experiment to contain exosome-positive markers HSP70, CD9, and CD63, but not the negative marker calnexin (Figure 1H). The average size of the exosomes from HMSMCs and HUVECs was 140.8 nm and 150 nm, respectively (Figure 1I). Immunofluorescence images demonstrated that red fluorescent Dil-labeled exosomes were absorbed by HMSMCs and HUVECs (Figure 1J–M), indicating there is exosome-mediated communication between the two major cell types in the myometrium.

### 2.2. Proteomic and miRNA Profiles of Exosomes in TNL and TL Myometrium

**Figure 2:**
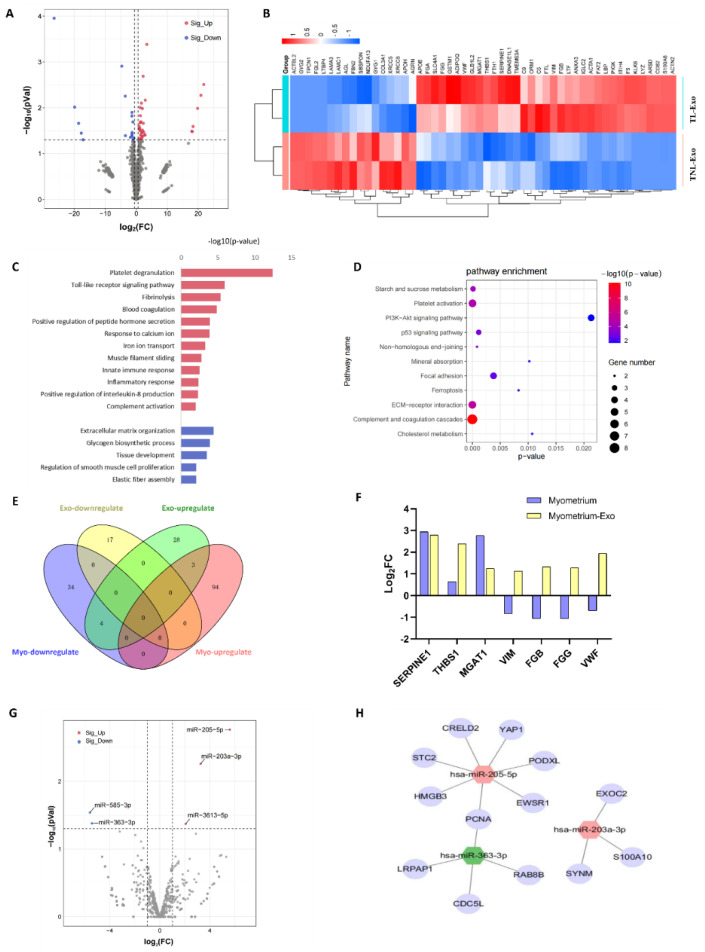
MicroRNA and proteomic profiles of exosomes in term non-labor and term labor myometrium. (**A**) Volcano plots for TNL (*n* = 2) vs. TL (*n* = 2) women. Significantly upregulated and downregulated proteins (adjusted *p* < 0.05) with a log_2_ fold change (FC) ±0.58 are represented by red and blue dots, respectively. Differentially expressed proteins that did not reach statistical significance are represented by gray dots. (**B**) Hierarchical clustering analysis of the screened DE proteins in the myometrium-derived exosome. (**C**) Bar diagram of the biology progress GO term clusters of DE proteins. (**D**) Bubble diagram of significantly enriched pathways for DE proteins. (**E**) Venn diagram of the DE gene set identified in exosome and tissue proteome. (**F**) Histogram of overlapping DE protein in the exosome and tissue. (**G**) Volcano plots for DE miRNAs of myometrial tissue exosomes in TNL and TL women. (**H**) Network of 2 upregulated miRNAs (hsa-miR-203a-3p and hsa-miR-205-5p) and 1 downregulated miRNA (hsa-miR-363-3p) and its target gene. Myo, myometrium.

A total of 1518 proteins were quantified in the myometrial exosomes. Fifty-two differentially expressed (DE) proteins were identified, including 35 upregulated and 17 downregulated proteins in TL, compared with TNL exosomes (Figure 2A,B and Supplemental Table S1). According to the enrichment analysis of the Gene Ontology (GO) biological processes, TL exosomes were significantly enriched with respect to coagulation, immunity, and ion-related pathways. Specifically, the multiple upregulated enrichment terms included platelet degranulation, toll-like reporter signaling pathways, fibrinolysis, blood coagulation, positive regulation of peptide hormone secretion, response to calcium ion, iron ion transport, muscle filament sliding, innate immune responses, inflammatory responses, and positive regulation of IL-8 production. The downregulated proteins in the TL exosomes were involved in extracellular matrix organization, glycogen biosynthetic processes, tissue development, regulation of muscle cell proliferation, and elastic fiber assembly (Figure 2C and Supplemental Table S2). In the KEGG pathway analysis, DE proteins were enriched in complement, coagulation cascades, platelet activation, and the ECM signaling pathway (Figure 2D and Supplemental Table S3). Our previously developed proteomic dataset [16] identified 135 DE proteins, which included 97 upregulated and 38 downregulated proteins. A Venn diagram of DE proteins common to exosomes and the myometrium showed three DE proteins (SERPINE1, THBS1, and MGAT1) upregulated both in the TL exosomes and myometrium, and four DE proteins (VIM, FGB, FGG, and VWF) that were upregulated in the exosomes while being downregulated in the myometrial tissues (Figure 2E,F).

A miRNA sequencing of the exosomes from the TNL and TL myometrium was also performed, and compared with the TNL group, three miRNAs (miR-203a-3p, miR-205-5p, and miR-3613-5p) were significantly upregulated and two miRNAs (miR-363-3p and miR-585-3p) were significantly downregulated in the TL myometrium (Figure 2G and Supplemental Table S4). The targets of these five miRNAs were predicted, then overlapped with the DE genes identified in the myometrium of the TNL and TL. A total of 13 genes (CRELD2, EWSR1, HMGB3, PCNA, PODXL, STC2, YAP1, CDC5L, LRPAP1, RAB8B, EXOC2, S100A10, and SYNM) were finally considered as the potential targets of the three miRNAs (miR-203a-3p, miR-205-5p, and miR-363-3p), which might establish the regulatory network between the exosomes and cells in the myometrium (Figure 2H). 

### 2.3. Cell-Specific Secreted miRNAs in the Tissue Exosomes

**Figure 3:**
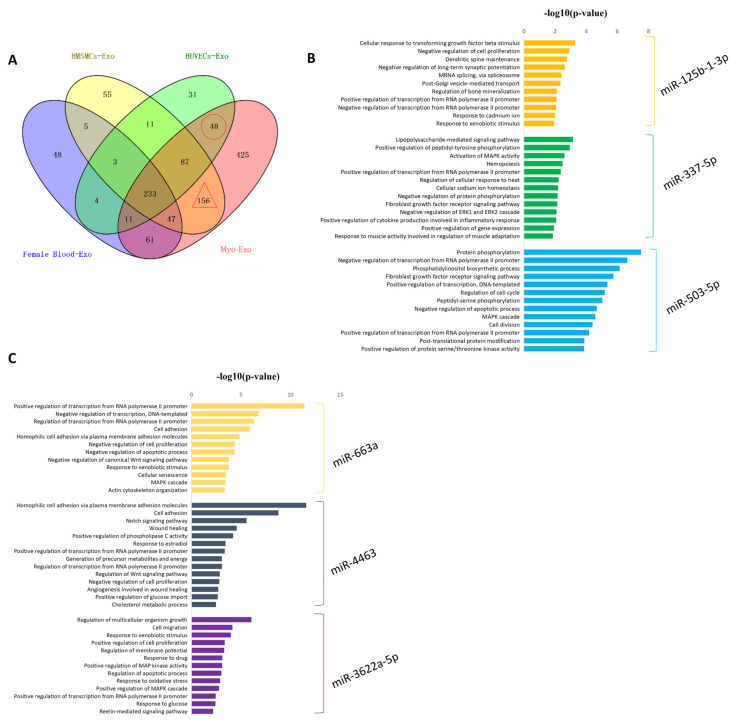
Cell-specific secreted miRNAs in tissue exosomes. (**A**) Venn diagram of the proteins identified in HMSMC, HUVEC, and female blood- and male blood-Exo. (**B**) Top 3 miRNAs with the highest expression levels in HMSMCs-specific miRNAs. (**C**) Top 3 miRNAs with the highest expression levels in HUVECs-specific miRNAs.

To explore the miRNAs specifically secreted by the HMSMCs and HUVECs, we compared the four groups of miRNA-seq data from the exosomes derived from the myometrium, HMSMCs, HUVECs, and female blood. The results revealed 156 miRNAs were specifically produced by HMSMCs (Figure 3A and Supplemental Table S5), of which the top three with the highest expression levels, miR-125b-1-3p, miR-337-5p, and miR-503-5p, showed their targets enriched the biological processes involved in transcriptional regulation, protein modification, and cellular responses (Figure 3B and Supplemental Table S6). Additionally, there were 48 miRNAs that were specifically secreted by HUVECs (Figure 3A and Supplemental Table S5), the top three with the highest expression levels, miR-663a, miR-4463, and miR-3622a-5p, were mainly related to the functions of actin cytoskeleton organization, cell proliferation, and apoptosis, metabolism, and oxidative stress (Figure 3C and Supplemental Table S7). These results indicate that the cargo of the exosomes from different myometrial cells were different, and these specifically secreted miRNAs might be key molecules that participate in the regulation of myometrial functions, which need further investigation.

### 2.4. Myometrial Contraction-Specific Biomarkers among Exosomal Proteins

**Figure 4:**
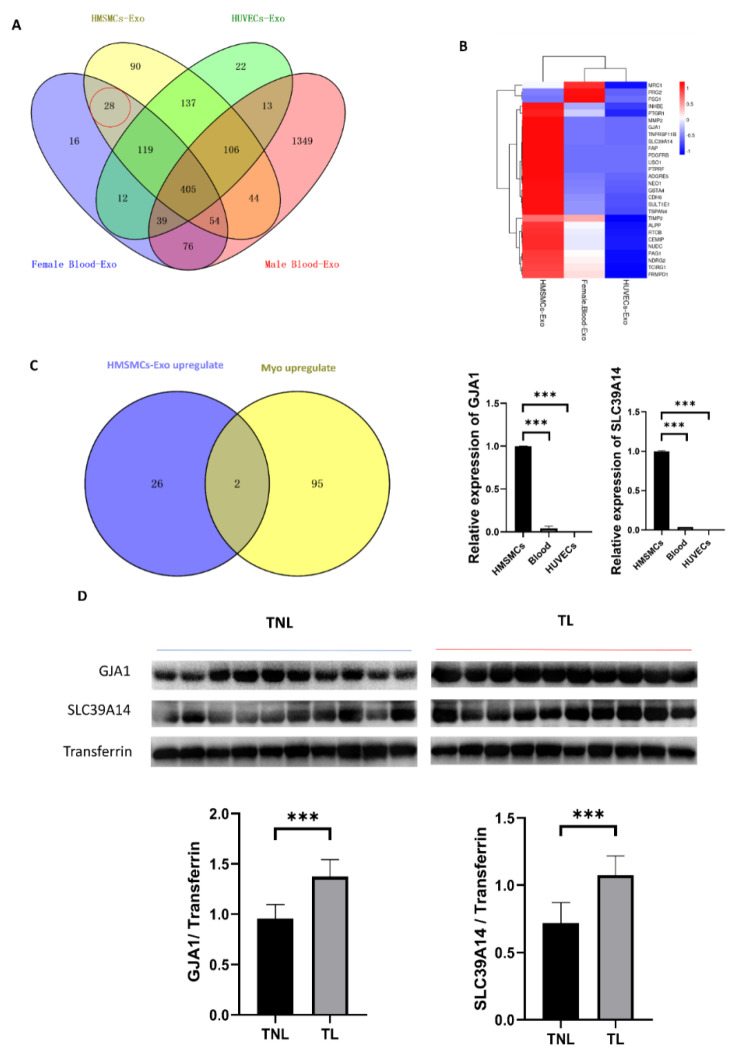
Myometrial contraction-specific biomarkers among exosomal proteins. (**A**) Venn diagram of miRNAs identified in HMSMC, HUVEC, and female blood- and male blood-Exo. (**B**) Heatmaps of the expression of the proteins identified in HMSMC, HUVEC, and female blood-Exo. (**C**) Venn diagram of proteins upregulated in HMSMCs-Exo and laboring myometrium, and GJA1 as well as SLC39A14 are the only two overlapping proteins and confirmed using qPCR. (**D**) GJA1 and SLC39A14 were upregulated in the peripheral blood of TL women; *n* = 10. *** *p* < 0.001.

To explore potential biomarkers of myometrial contraction for clinical detection, we performed proteomic analysis with protein mass spectrometry of the exosome derived from HMSMCs, HUVECs, and female peripheral blood (Supplemental Table S8). Proteins of the exosomes in healthy male peripheral blood [17] were excluded to identify biomarkers that are specifically secreted by myometrium. Twenty-eight common proteins were identified in HMSMCs and female blood exosomes (Figure 4A,B). The overlap of 28 upregulated proteins in HMSMC and 97 upregulated proteins in myometrial exosomes during labor showed that GJA1 (gap junction protein alpha 1) and SLC39A14 (solute carrier family 39 member 14) were increased during labor (Figure 4C). Peripheral blood is a common sample used for clinical detection, so we collected peripheral blood from TNL and TL women, verified the high expression in laboring peripheral blood (Figure 4D). GJA1 and SLC39A14 could be myometrial contraction-specific biomarkers used for clinical detection.

## 3. Discussion

Exosomes isolated from skeletal muscle tissue have been reported [18,19]; however, research on the uterine myometrial exosomes is lacking. In this study, we successfully isolated exosomes from the human myometrium and characterized its proteins and miRNA cargoes through liquid chromatography–tandem mass spectrometry (LC-MS/MS) and miRNA sequencing. Importantly, GJA1 and SLC39A14 were identified as blood biomarkers for myometrial activation, which are highly expressed in HMSMCs, and are also upregulated in the myometrium and peripheral blood during labor. Moreover, the regulatory network of DE miRNAs and their potential targets were elucidated. 

Exosomes are essential for cell–cell crosstalk, and previous research found that in fetal–maternal crosstalk, amnion epithelium-derived exosomes can be absorbed by maternal decidual and myometrial cells [20]. Our results revealed that both the exosomes from HMSMCs and HUVECs can be absorbed by the different cells. To the best of our knowledge, it is the first study to reprove the crosstalk between the main two types of cells in the myometrium. 

The inside cargo of the exosomes is considered to be a fingerprint, which changes with the state of the source cells or tissues [21,22,23]. Protein and miRNA, as the functional and regulated factors in cells, are the most concerned cargo in exosomes. Studies have reported that exosomes secreted by mesenchymal stem cells derived from human bone marrow, adipose tissue, and umbilical cords present different proteomic characteristics, involving the functions of regeneration, immune regulation, and damage repair, respectively [24]. The exosomes secreted by cardiac fibroblasts under hypoxic conditions contain more extracellular matrix-related proteins [25]. Endothelial cell-derived exosomes transferred atheroprotective signaling to smooth muscle cells via miR-143 and miR-145 [26]. In this study, we identified 52 proteins and 5 miRNAs differential expressed in the exosomes from human laboring and non-laboring myometrium, although the quantity of exosomes showed no difference. These DE proteins are mainly involved in coagulation, immunity, and ion-related pathways, which are the important events in the laboring process [27,28,29]. Combining the proteomics data of the myometrial exosomes and tissues, there are seven overlapped proteins, including three proteins (SERPINE1, THBS1, and MGAT1) that showed a consistent high expression level, and four proteins (VIM, FGB, FGG, and VWF) were upregulated in the exosomes while being downregulated in the tissues. This suggests that these proteins may be the mediators that bridge the connection between the cells in the myometrium. SERPINE1, FGB, FGG, and VWF are all key members in the coagulation pathway, THBS1 is an adhesion glycoprotein that mediates intercellular and cell–matrix interactions and MGAT1 is necessary for normal embryogenesis. The dynamic changes in these proteins between myometrial cells and exosomes may be the result of an intense contraction of myometrium during labor. Future work will be required to determine the mechanisms through which heterogeneous components are released into the contracted state of the myometrium.

Tissue-Exo was considered to be an appropriate candidate for biomarkers due to its high specificity. For example, an analysis of the tissue-Exo obtained from surgical margins and draining lymph nodes can be used to determine if the surgical extent is sufficient to clear tumor cells or if sentinel lymph nodes have been invaded [5]. HIST family proteins and METTL1 were overexpressed and specifically expressed in lung tumor tissues, respectively, and these biomarkers can be used for liquid biopsy [30]. In our study, we identified GJA1 and SLC39A14 as potential biomarkers of myometrial contraction because these two proteins are specifically present in female peripheral blood and are highly expressed in HMSMCs and are upregulated in the laboring myometrium. GJA1 is a well-known myometrial contraction associated with proteins [31]. SLC39A14 is a zinc transporter related to the cellular uptake of iron, manganese, and cadmium [32,33]. Subsequent peripheral blood tests confirmed the upregulated expression of GJA1 and SLC39A14 during labor, which are expected to become feasible markers for determining uterine activity. This is a primary result, and clinical application still has a long way to go in future research.

In conclusion, we successfully isolated exosomes from the human laboring myometrium, and provided a proteomic and miRNA profile of the exosomes. Our findings provide new insights into intercellular communication and novel contraction biomarkers in the myometrium.

## 4. Materials and Methods

### 4.1. Study Samples

For this study, myometrial tissues were collected from singleton, TNL, or TL women undergoing cesarean delivery at the Guangzhou Women and Children’s Medical Center (clinical characteristics are shown in Supplemental Table S9). TL was defined as having regular contractions and cervix dilations between 37 to 42 weeks’ gestation. The indication for the cesarean delivery was breech presentation or maternal request, with no medical reason. Exclusion criteria included the following: (1) complications including hypertensive disorders of pregnancy, gestational diabetes, and other diseases; (2) abnormal labor including uterine inertia or prolonged labor; and (3) fetal and placental abnormality including fetal distress, macrosomia, or malformation, placenta previa, placental abruption, or other conditions. Patient recruitment for this study was done through written informed consent and sample collection was approved by the ethics committee of Guangzhou Women and Children medical center (no. 201915401). Tissue fragments, obtained from the upper edge of lower segment uterine incision, were immediately frozen in liquid nitrogen at the time of cesarean delivery and stored at −80 °C.

### 4.2. Cell Culture

Primary HMSMCs were isolated as previous descried [34]. HMSMCs were cultured with Dulbecco’s Modified Eagle Medium (DMEM) (catalog #C11995500BT, Thermo Fisher Scientific, Waltham, MA, USA) containing 10% fetal bovine serum (FBS) (catalog #10099-141, Gibco, Grand Island, NY, USA ) and 1% penicillin-streptomycin (10,000 U/mL, catalog #15140122, Gibco, Grand Island, NY, USA). HUVECs were purchased from the American Type Culture Collection (ATCC, Washington, DC, NW, USA) and were cultured with DMEM (catalog #C11995500BT, Thermo Fisher Scientific, Waltham, MA, USA) containing 10% FBS (catalog #10099-141, Gibco, Grand Island, NY, USA) and 1% penicillin-streptomycin (10,000 U/mL, catalog #15140122, Gibco, Grand Island, NY, USA). Exosome-free FBS (catalog #EXO-FBS-50A-1, SBI, Pittsburgh, PA, USA)) was used in all of the exosome-related experiments.

### 4.3. Exosome Isolation from Myometrial Tissue

Extracellular vesicles were separated from the tissue, as described previously [35]. The dissociation mixture was based on the Miltenyi Human Tumor Dissociation Kit (catalog #130-095-929, Miltenyi Biotec, Bergisch Gladbach, Germany). According to the manufacturer‘s instructions, dissociation mix containing 2.2 mL RPMI, 100 μL enzyme H, 50 μL enzyme R, and 12.5 μL enzyme A was prepared immediately before starting. Briefly, a small frozen piece of tissue was weighed to 200 mg, and was sliced on dry ice and incubated in the dissociation mixture for 10–15 min at 37 °C. The dissociated tissue was carefully filtered twice through a 70 μm filter to remove the residual tissue. The suspension was then centrifuged at 300× *g* for 10 min at 4 °C, and the supernatant was centrifuged again at 2000× *g* and 4 °C for 10 min. Then, the cell-free supernatant was centrifuged at 10,000× *g* and 4 °C for 20 min and was filtered through a 0.22 μm filter for further reducing the cell debris. The collected suspension was processed by ultracentrifugation at 150,000× *g* and 4 °C for 2 h. The pellet was resuspended in 1 mL PBS and was further purified using Exosupur columns (Echobiotech, Beijing, China). Fractions were concentrated to 200 μL at a 100 kDa molecular weight cut-off using Amicon Ultra spin filters (Merck, Darmstadt, Germany).

### 4.4. Exosome Isolation from Plasma

Individual peripheral blood samples were placed in EDTA tubes following conventional venipuncture. After centrifugation at 3000× *g* and 4 °C for 15 min, plasma was aspirated and was stored at −80 °C. The ultracentrifugation method was optimized according to a previous study [36]. After thawing at 37 °C, plasma samples were centrifugated at 3000× *g* for 15 min to remove cell debris. Then, the supernatant was diluted with the seven-fold volume of PBS, centrifuged at 13,000× *g* for 30 min, and was processed through a 0.22-μm filter to remove large particles. The supernatant was ultracentrifuged using a P50A72-986 rotor (CP100NX; Hitachi, Brea, CA, USA) at 100,000× *g* and 4 °C for 2 h to pellet exosomes. The pellet was resuspended in PBS and was centrifuged again at 100,000× *g* and 4 °C for 2 h. After washing with PBS, the exosome pellet was re-suspended in 100 µL of PBS. The exosomes were isolated by size exclusion chromatography, as described previously, with minor modifications [36]. Briefly, 1 mL of 0.8 μm filtered blood plasma was 1.5-fold diluted with PBS and further purified using Exosupur columns (Echobiotech, Beijing, China). The samples were then eluted with 0.1 M PBS and 2 mL eluate fractions were collected, according to the manufacturer’s instructions. Fractions were concentrated to 200 μL with a 100 kDa molecular weight cut-off using Amicon Ultra spin filters (Merck, Darmstadt, Germany).

### 4.5. Exosome Isolation from Cell Supernatant

After culturing under hypoxia or normoxia, the conditioned medium was carefully collected and sequentially centrifuged at 300× *g* for 10 min, 2000× *g* for 10 min (fixed rotor; Thermo Fisher Scientific, Waltham, MA, USA), and 10,000× *g* for 30 min (JA25.5 rotor, Beckman Optima JXN26; Beckman Coulter, Brea, CA, USA) at constant 4 °C to remove dead cells and cell debris. The supernatant was then collected for ultracentrifugation at 100,000× *g* and 4 °C for 70 min (70Ti rotor, Beckman Optima XPN100; Beckman Coulter, Brea, CA, USA). The pellet was re-suspended in 1× PBS and was then centrifuged again at 100,000× *g* and 4 °C for 70 min (sw41Ti rotor, Beckman Optima XPN100; Beckman Coulter, Brea, CA, USA). The pellet was then re-suspended in 1× PBS and was stored at −80 °C.

### 4.6. NTA

ZetaView PMX 110 (Particle Metrix, Meerbusch, Germany), a nanoparticle tracking video microscope, was used for the NTA. The exosomes were appropriately diluted with PBS (dilution factor: 400–1000), and size distribution and concentration were assessed under 405 nm emission light. Using fluorescence to scan the different positions of the sample pool 11 times and by capturing the Brownian motion trajectory of the nanoparticles, they were counted and their size was calculated with Origin 2018 (OriginLab Corporation, Northampton, MA, USA) and GraphPad Prism 8 (GraphPad Software, LLC, San Diego, CA, USA). 

### 4.7. Transmission Electron Microscopy

The exosome shape was determined using a transmission electron microscope (H7650; Hitachi, Tokyo, Japan). The exosome pellet solution (10 µL) was loaded on a copper mesh and was incubated at room temperature for 10 min, followed by washing with sterile distilled water; the excess liquid was discarded. The solution was then subjected to negative staining with 10 µL 2% uranyl acetate for 1 min. After absorbing the floating liquid with filter paper, it was dried under an incandescent lamp for 2 min and was imaged with 80 kV.

### 4.8. Immunofluorescence

Immunofluorescence was performed to identify the uptake of exosomes inside the HMSMCs and HUVECs. Fluorescently labeled exosomes were prepared using a Cell Plasma Membrane Staining Kit with DiI (catalog #C1991S, Beyotime, Shanghai, China) with the following steps: (1) prepare Dil working fluid refer to instruction manual; (2) incubate exosomes with Dil working fluid for 10–20 min in the dark; (3) transfer the working solution containing exosomes to a 12.5 mL ultracentrifuge tube and centrifuge at 190,000× *g* for 2 h; (4) resuspend the pellet in culture medium. Cells in the 24-wells plates were fixed with 4% Paraformaldehyde (PFA) (catalog #BL539A, Biosharp Co., Ltd., Guangzhou, China) for 30 min and washed three times with PBS, 5 min each time, and then treated with PBS with 0.3% TritonX-100 (catalog #0219485480, MPBio, Irvine, CA, USA) for 15 min, followed by a treatment of goat serum to block non-specific antigens for 1 h at room temperature. The cells were incubated with primary antibodies to α-smooth muscle actin (HMSMCs marker; 6.82 μg/mL; catalog #ab7817-100, Abcam, Cambridge, UK) and CD34 (HUVECs marker; 1:200; catalog #ab8129-100, Abcam, Cambridge, UK) over night at 4 °C. After washing the cells several times with PBS, the cells were incubated with Alexa 488 Goat anti-Mouse secondary antibody diluted 1:250 in PBS (catalog #ab15077, Abcam, Cambridge, UK) for 1 h in the dark. Mounting medium with 4′, 6-diamidino-2-phenylindole (DAPI) (catalog #G1236-100T, Servicebio, Guangzhou, China) was added to the wells. The cells were imaged via fluorescence microscopy (DMi8, Leica, Wetzlar, Germany) and analyzed using Leica Application Suite X.

### 4.9. Western Blotting

The proteins of the HMSMCs and HUVECs were extracted with a RIPA lysis buffer (catalog #P0013B, Beyotime). Samples suspended in RIPA were centrifuged at 15,000× rpm for 5 min and the supernatant was collected. Plasma protein was extracted according to the kit protocol (catalog#BB-3137, BestBio, Nanjing, China). The protein concentration of the cell samples and exosomes was measured through a bicinchoninic acid assay (BCA) kit (catalog#23227, Thermo Scientific, Waltham, MA, USA). The protein samples and exosome fractions were loaded in SDS-PAGE Precast Gels (catalog # SS0110, Sorfa, Shanghai, China), separated by electrophoresis, and transferred onto polyvinylidene difluoride membranes (catalog#IPVH00010, Millipore, Merck). After being blocked with 5% skim milk and washed with 0.1% Tris-buffered saline with Tween-20, the protein was probed with primary antibodies listed in Table 1. On the next day, the membrane was probed with secondary antibody at room temperature for 2 h. The proteins were quantified via a Bio-Rad’s ChemiDoc XRS+ System and imaged by Image Lab Software. Tsg101, CD9, Hsp70, and CD 63 were used as exosome positive markers and Calnexin was used as the exosome negative marker. High expressed proteins in the HMSMCs-exosome, GJA1 (one of myometrial contraction associated proteins) and SLC39A14 (a zinc transporter), were verified in the human plasma. The relative expression levels of the plasma proteins were normalized to that of Transferrin. The Western blotting antibodies used are listed in Table 1. 

### 4.10. Label-free Quantitative Proteomics

#### 4.10.1. Protein Extraction and Trypsin Treatment

Exosome samples were lysed with a lysis buffer (containing 100 mM NH4HCO3, 6M Urea, and 0.2% SDS) and sonicated extensively on ice. After centrifuging the lysate at 12,000× *g* for 15 min at 4 °C, the supernatant was collected and transferred to a clean tube. The samples were reduced with 10 mM DTT for 1 h at 56 °C and alkylated with iodoacetamide for 1 h at room temperature in the dark, followed by mixing with 4 volumes of acetone and being incubated at −20 °C for 2 h. After centrifugation, the pellet was washed with cold acetone and collected with 0.1 M TEAB and 6 M urea. The protein concentration of the samples was calculated from a standard protein curve using the PierceTM BCA Protein Assay Kit (catalog# 23,225, Thermo Scientific, Waltham, MA, USA), according to the manufacturer’s instructions.

Each sample was added with 3 μL trypsin (1 μg/μL) and 500 μL TEAB buffer (100 mM), then digested overnight at 37 °C. The samples were added with 1% formic acid and centrifuged at 12,000× *g* for 5 min at room temperature. The centrifuged sample supernatant was slowly loaded onto a C18 desalting column, washed with a wash buffer (0.1% formic acid and 4% acetonitrile) and eluted by an elution buffer (0.1% formic acid and 75% acetonitrile). The eluents were lyophilized for further experiments. 

#### 4.10.2. LC-MS/MS Analysis

The lyophilized samples were dissolved in 0.1% formic acid solution (solvent A), and injected into a C18 Nano-Trap column (2 cm × 75 μm, 3 μm). The peptides were separated in an analytical column (15 cm × 150 μm, 1.9 μm) with a mobile phrase of 0.1% formic in 80% acetonitrile (solvent B). The samples were eluted by increasing the concentration of solvent B from 6% to 100% over 60 min, maintaining a flow rate of 600 nL/min. The separated peptides were injected into Nanospray Flex ESI with spray voltage of 2.3 kV and were analyzed with an Orbitrap Exploris 480 (Thermo Fisher, Waltham, MA, USA). The UniProt database (http://www.uniprot.org; release-2021_04/) was used to search the raw data for the MS assays. Carbamate was set as a fixed modification. Oxidation of methionine (M) and acetylation of the N-terminus were set as the variable modifications. The label-free proteins were quantified using Proteome Discoverer software version 2.2, one unique peptide for cut-off, with the following parameters: Precursor ion mass tolerance (±15 ppm), fragment ion mass tolerance (±0.02 Da), and Max Missed Cleavages (2). The differential expressed proteins were analyzed with limma package, log_2_(abundance + 1) treated matrix was used to calculate the *p* value and log_2_FC.

### 4.11. Exosomal MiRNA Analysis

#### 4.11.1. miRNA Isolation and Sequencing

The total RNA was extracted and purified from the exosome using the miRNeasy Mini kit (catalog#217004, Qiagen, Düsseldorf, Germany) according to the kit instruction. The RNA concentration and purity were evaluated using the RNA Nano 6000 Assay Kit of the Agilent Bioanalyzer 2100 System (Agilent Technologies, Palo Alto, CA, USA). Agilent 2100 (an RNA sample quality control analysis instrument, Agilent Technologies, Palo Alto, CA, USA) was used for the qualitative analysis.

A total amount of 500 ng RNA per sample was used as the input material for sequencing libraries using the QIAseq miRNA Library Kit (Qiagen, Frederick, MD, USA), according to manufacturer’s instructions, and an index code was added to attribute the sequence to label each sample. Subsequently, the library quality was assessed on an Agilent Bioanalyzer 2100 and qPCR. Index-encoded samples were clustered on the acBot Cluster Generation System using the TruSeq PE Cluster Kitv3-cBot-HS (Illumina, San Diego, CA, USA) according to the manufacturer’s instructions. After cluster generation, the library preparation was sequenced using the Illumina Hiseq platform and the paired-end reads were generated.

#### 4.11.2. Bioinformatic Analysis of MiRNA

The Clean Reads sequences were aligned with the Silva database, GtRNAdb database, Rfam database, and Repbase database by using the Bowtie tools soft, filter ribosomal RNA (rRNA), transfer RNA (tRNA), small nuclear RNA (snRNA), small nucleolar RNA (snoRNA), and other ncRNA and repeats. The remaining reads were used to identify known miRNA and new miRNA predicted by comparing them with known miRNAs from the miRbase and Human Genome (GRCh38), respectively. The read counts for each miRNA were taken from the mapping results and TPM was calculated. The comparison between the two groups of repeated samples was analyzed using the limma R package [37]. For the differential expression analysis between two samples, we calculated the significance of the differences using the Significance A/B method [38]. The screening criteria were |log_2_(FC)| ≥ 0.5849625; *p*-value ≤ 0.05. The targets of miRNA were predicted using the EVmiRNA database (http://bioinfo.life.hust.edu.cn/EVmiRNA#!/; URL (accessed on 7 April 2022)).

### 4.12. GO and KEGG Pathway Enrichment Analysis

The GO and KEGG pathway enrichment analysis of the target genes of miRNAs was analyzed by DAVID (https://david.ncifcrf.gov/; release-2021_04/). The screening criteria were *p*-value ≤ 0.05.

### 4.13. RT-qPCR

RT-qPCR was performed to confirm the gene expression of GJA1 and SLC39A14 in the HMSMC-exosome. mRNA was reverse transcribed using PrimeScript RT Master Mix (catalog#RR036B, TAKARA, Otsu, Shiga, Japan). qPCR was using TB Green Premix Ex Taq II (catalog#RR820B, TAKARA, Otsu, Shiga, Japan) and performed via StepOnePlus Real-Time PCR System (Applied Biosystems, Foster City, CA, USA). The qPCR conditions were as follows: 95 °C for 10 min, 40 cycles at 95 °C for 15 s, and 60 °C for 1 min. The relative expression levels of mRNA were normalized to that of alpha smooth muscle actin (ACTB) and were calculated with the 2^−ΔΔCT^ method. The primers are listed in Table 2.

### 4.14. Statistics Analysis

Data for continuous variables were presented as mean ± SEM. Statistics were calculated with GraphPad Prism 8.0. The statistical significance of the test results was evaluated by one-way ANOVA. A *p* value of less than 0.05 was considered statistically significant.

## Figures and Tables

**Table 1:** Information of the antibodies used in this study.

Antibody Name	Catalog No.	Company	Concentration
Tsg101	Ab125011	Abcam, Cambridge, UK	1:1000
CD9	60232-I-Ig	Proteintech, Rosemont, IL, USA	1:1000
HSP70	ab181606	Abcam, Cambridge, UK	1:1000
Calnexin	10427-2-AP	Proteintech, Rosemont, IL, USA	1:500
CD9	ab198702	Abcam, Cambridge, UK	1:2000
CD63	ab134045	Abcam, Cambridge, UK	1:1000
HSP70	ab181606	Abcam, Cambridge, UK	1:2000
Calnexin	ab133615	Abcam, Cambridge, UK	1:1000
GJA1	ab217676	Abcam, Cambridge, UK	1:1000
SLC39A14	A10413	Affinity Biosciences, Cincinnati, OH, USA	1:1000
Transferrin	ab277635	Abcam, Cambridge, UK	1:1000

**Table 2:** The sequences of the primers of human target genes.

Gene	Sequence (5′-3′)	
GJA1	F: GGAGATGAGCAGTCTGCCTTTC	R: TGAGCCAGGTACAAGAGTGTGG
SLC39A14	F: CTGGACCACATGATTCCTCAGC	R: AGAGTAGCGGACACCTTTCAGC
ACTB	F: CACCATTGGCAATGAGCGGTTC	R: AGGTCTTTGCGGATGTCCACGT

Abbreviations: F: forward primer; R: reserve primer.

## Data Availability

All the data generated or analyzed during the current study are available in the supplementary information files or from the corresponding author on reasonable request.

## Supplementary Materials

The supporting information can be downloaded at: https://www.mdpi.com/article/10.3390/ijms232012343/s1.
